# Supplementary material for: Evaluating the effects of community-based programs on viral rebound and viral suppression among HIV-positive orphaned and vulnerable children receiving antiretroviral treatment: Findings from the ACHIEVE project in Tanzania
Source: PLoS One. 2026 May 15;21(5):e0349141. doi: 10.1371/journal.pone.0349141 (PMC13178892; doi:10.1371/journal.pone.0349141)
Supplement: S1 File — S1 Table. Coverage of ACHIEVE project services among CLHIV as of July 15th, 2023. S2 Table. Factors associated with viral rebound at follow-up among 21,448 CLHIV who had undetectable viral load at baseline in Tanzania (ACHIEVE project interventions analysed as separate variables). S3 Table. Factors associated with undetectable viral load at follow-up among 4,809 CLHIV who had detectable viral load at baseline in Tanzania (ACHIEVE project interventions analysed as separate variables). S4 Table. Factors associated with viral rebound at follow-up among 21,448 CLHIV who had undetectable viral load at baseline in Tanzania (ACHIEVE project interventions reduced into a single binary variable). S5 Table. Factors associated with undetectable viral load at follow-up among 4,809 CLHIV who had detectable viral load at baseline in Tanzania (ACHIEVE project interventions reduced into a single binary variable). (ZIP) [file pone.0349141.s001.zip › Supporting information/S1 Table 1.docx]

| **S1 Table 1. Coverage of ACHIEVE project services among CLHIV as of July 15th, 2023** | |
| --- | --- |
|  | **Coverage of ACHIEVE project services among CLHIV (n = 26,257)** |
| CLHIV whose caregivers were participants in WORTH Yetu economic strengthening | 5,051 (19.2%) |
| CLHIV linked to teen/paediatric clubs | 22,976 (87.5%) |
| CLHIV in households that received health insurance (iCHF) | 6,861 (26.1%) |
| CLHIV received adherence support provided by CCWs) | 26,110 (99.4%) |
| CLHIV with good adherence to ART* | 24,275/24,337 (99.7%) |
| CLHIV received case management visits by CCWs) | 26,047 (99.2%) |
| *Data not available for all CLHIV | |
