# Supplementary material for: Evaluating the effects of community-based programs on viral rebound and viral suppression among HIV-positive orphaned and vulnerable children receiving antiretroviral treatment: Findings from the ACHIEVE project in Tanzania
Source: PLoS One. 2026 May 15;21(5):e0349141. doi: 10.1371/journal.pone.0349141 (PMC13178892; doi:10.1371/journal.pone.0349141)
Supplement: S1 File — S1 Table. Coverage of ACHIEVE project services among CLHIV as of July 15th, 2023. S2 Table. Factors associated with viral rebound at follow-up among 21,448 CLHIV who had undetectable viral load at baseline in Tanzania (ACHIEVE project interventions analysed as separate variables). S3 Table. Factors associated with undetectable viral load at follow-up among 4,809 CLHIV who had detectable viral load at baseline in Tanzania (ACHIEVE project interventions analysed as separate variables). S4 Table. Factors associated with viral rebound at follow-up among 21,448 CLHIV who had undetectable viral load at baseline in Tanzania (ACHIEVE project interventions reduced into a single binary variable). S5 Table. Factors associated with undetectable viral load at follow-up among 4,809 CLHIV who had detectable viral load at baseline in Tanzania (ACHIEVE project interventions reduced into a single binary variable). (ZIP) [file pone.0349141.s001.zip › Supporting information/S1 Table 2.docx]

| **S1 Table 2. Factors associated with viral rebound at follow-up among 21,448 CLHIV who had undetectable viral load at baseline in Tanzania (ACHIEVE project interventions analysed as separate variables)** | | | | |
| --- | --- | --- | --- | --- |
|  | **adjusted Odds Ratio (aOR)** | **Lower 95% confidence limit** | **Upper 95% confidence limit** | ***p*-value** |
| **ART regimen** |  |  |  |  |
| DTG-based | 1.000 | — | — | — |
| Other regimens | 1.378 | 1.109 | 1.712 | 0.004 |
| **Duration in the ACHIEVE project** |  |  |  |  |
| <6 months | 1.000 | — | — | — |
| 6-11 months | 0.779 | 0.369 | 1.644 | 0.51 |
| 12+ months | 0.718 | 0.342 | 1.505 | 0.38 |
| **CLHIV's caregiver participates in WORTH Yetu?** |  |  |  |  |
| No | 1.000 | — | — | — |
| Yes | 0.953 | 0.859 | 1.057 | 0.36 |
| **CLHIV linked to teen/paediatric clubs** |  |  |  |  |
| No | 1.000 | — | — | — |
| Yes | 0.836 | 0.743 | 0.941 | 0.003 |
| **Health insurance (iCHF)** |  |  |  |  |
| No | 1.000 | — | — | — |
| Yes | 0.762 | 0.690 | 0.842 | < 0.001 |
| **CLHIV sex** |  |  |  |  |
| Female | 1.000 | — | — | — |
| Male | 0.973 | 0.898 | 1.054 | 0.50 |
| **CLHIV age** |  |  |  |  |
| <5 years | 1.000 | — | — | — |
| 5-9 years | 1.001 | 0.820 | 1.221 | 1.00 |
| 10-14 years | 1.059 | 0.852 | 1.316 | 0.604 |
| 15-17 years | 1.010 | 0.810 | 1.260 | 0.93 |
| **CLHIV's caregiver age** |  |  |  |  |
| 18-29 years | 1.000 | — | — | — |
| 30-39 years | 0.885 | 0.788 | 0.993 | 0.038 |
| 40-49 years | 0.897 | 0.805 | 1.001 | 0.052 |
| 50-59 years | 0.835 | 0.717 | 0.973 | 0.02 |
| 60+ years | 0.885 | 0.741 | 1.057 | 0.18 |
| **CLHIV school attendance status** |  |  |  |  |
| Not attending | 1.000 | — | — | — |
| Attending | 1.114 | 0.968 | 1.283 | 0.13 |
| **Level of household hunger** |  |  |  |  |
| Little to no hunger | 1.000 | — | — | — |
| Moderate hunger | 1.051 | 0.934 | 1.182 | 0.407 |
| Severe hunger | 1.066 | 0.838 | 1.357 | 0.601 |
| **Place of residence** |  |  |  |  |
| Rural | 1.000 | — | — | — |
| Urban | 1.243 | 1.137 | 1.358 | < 0.001 |
| **Family size** |  |  |  |  |
| 2-3 people | 1.000 | — | — | — |
| 4-6 people | 0.883 | 0.805 | 0.968 | 0.008 |
| 7+ people | 0.912 | 0.660 | 1.261 | 0.58 |
| **Caregiver sex** |  |  |  |  |
| Female | 1.000 | — | — | — |
| Male | 1.011 | 0.928 | 1.102 | 0.80 |
| **Caregiver education** |  |  |  |  |
| Never attended | 1.000 | — | — | — |
| Primary | 1.193 | 1.063 | 1.339 | 0.003 |
| Secondary+ | 1.326 | 1.064 | 1.654 | 0.012 |
| **ART change in the last 6 months** |  |  |  |  |
| No | 1.000 | — | — | — |
| Yes | 1.153 | 1.031 | 1.288 | 0.012 |
| Constant | 0.193 | 0.089 | 0.419 | < 0.001 |
