# Supplementary material for: Evaluating the effects of community-based programs on viral rebound and viral suppression among HIV-positive orphaned and vulnerable children receiving antiretroviral treatment: Findings from the ACHIEVE project in Tanzania
Source: PLoS One. 2026 May 15;21(5):e0349141. doi: 10.1371/journal.pone.0349141 (PMC13178892; doi:10.1371/journal.pone.0349141)
Supplement: S1 File — S1 Table. Coverage of ACHIEVE project services among CLHIV as of July 15th, 2023. S2 Table. Factors associated with viral rebound at follow-up among 21,448 CLHIV who had undetectable viral load at baseline in Tanzania (ACHIEVE project interventions analysed as separate variables). S3 Table. Factors associated with undetectable viral load at follow-up among 4,809 CLHIV who had detectable viral load at baseline in Tanzania (ACHIEVE project interventions analysed as separate variables). S4 Table. Factors associated with viral rebound at follow-up among 21,448 CLHIV who had undetectable viral load at baseline in Tanzania (ACHIEVE project interventions reduced into a single binary variable). S5 Table. Factors associated with undetectable viral load at follow-up among 4,809 CLHIV who had detectable viral load at baseline in Tanzania (ACHIEVE project interventions reduced into a single binary variable). (ZIP) [file pone.0349141.s001.zip › Supporting information/S1 Table 3.docx]

| **S1 Table 3. Factors associated with undetectable viral load at follow-up among 4,809 CLHIV who had detectable viral load at baseline in Tanzania (ACHIEVE project interventions analysed as separate variables)** | | | | |
| --- | --- | --- | --- | --- |
|  | **adjusted Odds Ratio (aOR)** | **Lower 95% confidence limit** | **Upper 95% confidence limit** | ***p*-value** |
| **ART regimen** |  |  |  |  |
| DTG-based | 1.000 | — | — | — |
| Other regimens | 0.924 | 0.684 | 1.248 | 0.606 |
| **Duration in the ACHIEVE project** |  |  |  |  |
| <6 months | 1.000 | — | — | — |
| 6-11 months | 1.757 | 0.588 | 5.254 | 0.31 |
| 12+ months | 1.459 | 0.497 | 4.285 | 0.49 |
| **CLHIV's caregiver participates in WORTH Yetu?** |  |  |  |  |
| No | 1.000 | — | — | — |
| Yes | 0.969 | 0.826 | 1.137 | 0.70 |
| **CLHIV linked to teen/paediatric clubs** |  |  |  |  |
| No | 1.000 | — | — | — |
| Yes | 1.267 | 1.047 | 1.532 | 0.015 |
| **Health insurance (iCHF)** |  |  |  |  |
| No | 1.000 | — | — | — |
| Yes | 1.235 | 1.056 | 1.444 | 0.008 |
| **CLHIV sex** |  |  |  |  |
| Female | 1.000 | — | — | — |
| Male | 0.952 | 0.840 | 1.079 | 0.44 |
| **CLHIV age** |  |  |  |  |
| <5 years | 1.000 | — | — | — |
| 5-9 years | 1.163 | 0.888 | 1.524 | 0.27 |
| 10-14 years | 1.146 | 0.844 | 1.557 | 0.38 |
| 15-17 years | 0.996 | 0.728 | 1.363 | 0.98 |
| **CLHIV's caregiver age** |  |  |  |  |
| 18-29 years | 1.000 | — | — | — |
| 30-39 years | 0.982 | 0.822 | 1.173 | 0.84 |
| 40-49 years | 1.162 | 0.978 | 1.381 | 0.089 |
| 50-59 years | 1.502 | 1.163 | 1.940 | 0.002 |
| 60+ years | 1.138 | 0.849 | 1.526 | 0.39 |
| **CLHIV school attendance status** |  |  |  |  |
| Not attending | 1.000 | — | — | — |
| Attending | 0.820 | 0.659 | 1.021 | 0.076 |
| **Level of household hunger** |  |  |  |  |
| Little to no hunger | 1.000 | — | — | — |
| Moderate hunger | 1.057 | 0.882 | 1.266 | 0.55 |
| Severe hunger | 1.156 | 0.796 | 1.678 | 0.45 |
| **Place of residence** |  |  |  |  |
| Rural | 1.000 | — | — | — |
| Urban | 0.799 | 0.696 | 0.918 | 0.002 |
| **Family size** |  |  |  |  |
| 2-3 people | 1.000 | — | — | — |
| 4-6 people | 0.994 | 0.863 | 1.146 | 0.94 |
| 7+ people | 1.063 | 0.637 | 1.773 | 0.82 |
| **Caregiver sex** |  |  |  |  |
| Female | 1.000 | — | — | — |
| Male | 0.972 | 0.850 | 1.112 | 0.68 |
| **Caregiver education** |  |  |  |  |
| Never attended | 1.000 | — | — | — |
| Primary | 0.924 | 0.765 | 1.117 | 0.42 |
| Secondary+ | 1.086 | 0.759 | 1.555 | 0.65 |
| **ART change in the last 6 months** |  |  |  |  |
| No | 1.000 | — | — | — |
| Yes | 0.954 | 0.796 | 1.144 | 0.61 |
| Constant | 1.472 | 0.476 | 4.549 | 0.502 |
