# Supplementary material for: Evaluating the effects of community-based programs on viral rebound and viral suppression among HIV-positive orphaned and vulnerable children receiving antiretroviral treatment: Findings from the ACHIEVE project in Tanzania
Source: PLoS One. 2026 May 15;21(5):e0349141. doi: 10.1371/journal.pone.0349141 (PMC13178892; doi:10.1371/journal.pone.0349141)
Supplement: S1 File — S1 Table. Coverage of ACHIEVE project services among CLHIV as of July 15th, 2023. S2 Table. Factors associated with viral rebound at follow-up among 21,448 CLHIV who had undetectable viral load at baseline in Tanzania (ACHIEVE project interventions analysed as separate variables). S3 Table. Factors associated with undetectable viral load at follow-up among 4,809 CLHIV who had detectable viral load at baseline in Tanzania (ACHIEVE project interventions analysed as separate variables). S4 Table. Factors associated with viral rebound at follow-up among 21,448 CLHIV who had undetectable viral load at baseline in Tanzania (ACHIEVE project interventions reduced into a single binary variable). S5 Table. Factors associated with undetectable viral load at follow-up among 4,809 CLHIV who had detectable viral load at baseline in Tanzania (ACHIEVE project interventions reduced into a single binary variable). (ZIP) [file pone.0349141.s001.zip › Supporting information/S1 Table 4.docx]

| **S1 Table 4. Factors associated with viral rebound at follow-up among 21,448 CLHIV who had undetectable viral load at baseline in Tanzania (ACHIEVE project interventions reduced into a single binary variable)** | | | | |
| --- | --- | --- | --- | --- |
|  | **adjusted Odds Ratio (aOR)** | **Lower 95% confidence limit** | **Upper 95% confidence limit** | ***p*-value** |
| **ART regimen type** |  |  |  |  |
| DTG-based | 1.000 | — | — | — |
| Other regimens | 1.382 | 1.113 | 1.717 | 0.003 |
| **Months project** |  |  |  |  |
| <6 months | 1.000 | — | — | — |
| 6-11 months | 0.777 | 0.368 | 1.641 | 0.508 |
| 12-23 months | 0.676 | 0.322 | 1.419 | 0.301 |
| **Number of ACHIEVE project interventions received** |  |  |  |  |
| None (0) | 1.000 | — | — | — |
| One or more (≥1) | 0.768 | 0.668 | 0.882 | < 0.001 |
| **CLHIV sex** |  |  |  |  |
| Female | 1.000 | — | — | — |
| Male | 0.971 | 0.896 | 1.052 | 0.47 |
| **CLHIV age** |  |  |  |  |
| <5 years | 1.000 | — | — | — |
| 5-9 years | 1.005 | 0.824 | 1.226 | 0.96 |
| 10-14 years | 1.068 | 0.859 | 1.326 | 0.55 |
| 15-17 years | 1.022 | 0.820 | 1.274 | 0.85 |
| **Caregiver age** |  |  |  |  |
| 18-29 years | 1.000 | — | — | — |
| 30-39 years | 0.849 | 0.758 | 0.951 | 0.005 |
| 40-49 years | 0.860 | 0.773 | 0.958 | 0.006 |
| 50-59 years | 0.796 | 0.685 | 0.925 | 0.003 |
| 60+ years | 0.849 | 0.711 | 1.013 | 0.069 |
| **CLHIV school attendance status** |  |  |  |  |
| Not attending | 1.000 | — | — | — |
| Attending school | 1.117 | 0.970 | 1.285 | 0.13 |
| **Level of household hunger** |  |  |  |  |
| Little to no hunger | 1.000 | — | — | — |
| Moderate hunger | 1.053 | 0.936 | 1.184 | 0.39 |
| Severe hunger | 1.062 | 0.835 | 1.351 | 0.62 |
| **Place of residence** |  |  |  |  |
| Rural | 1.000 | — | — | — |
| Urban | 1.231 | 1.128 | 1.345 | < 0.001 |
| **Family size** |  |  |  |  |
| 2-3 people | 1.000 | — | — | — |
| 4-6 people | 0.875 | 0.798 | 0.960 | 0.005 |
| 7+ people | 0.895 | 0.648 | 1.237 | 0.502 |
| **Caregiver sex** |  |  |  |  |
| Female | 1.000 | — | — | — |
| Male | 1.008 | 0.926 | 1.098 | 0.85 |
| **Caregiver education** |  |  |  |  |
| Never attended | 1.000 | — | — | — |
| Primary | 1.204 | 1.073 | 1.351 | 0.002 |
| Secondary+ | 1.347 | 1.081 | 1.680 | 0.008 |
| **ART change in the last 6 months** |  |  |  |  |
| No | 1.000 | — | — | — |
| Yes | 1.156 | 1.035 | 1.292 | 0.01 |
| Constant | 0.208 | 0.096 | 0.452 | < 0.001 |
