# Supplementary material for: Evaluating the effects of community-based programs on viral rebound and viral suppression among HIV-positive orphaned and vulnerable children receiving antiretroviral treatment: Findings from the ACHIEVE project in Tanzania
Source: PLoS One. 2026 May 15;21(5):e0349141. doi: 10.1371/journal.pone.0349141 (PMC13178892; doi:10.1371/journal.pone.0349141)
Supplement: S1 File — S1 Table. Coverage of ACHIEVE project services among CLHIV as of July 15th, 2023. S2 Table. Factors associated with viral rebound at follow-up among 21,448 CLHIV who had undetectable viral load at baseline in Tanzania (ACHIEVE project interventions analysed as separate variables). S3 Table. Factors associated with undetectable viral load at follow-up among 4,809 CLHIV who had detectable viral load at baseline in Tanzania (ACHIEVE project interventions analysed as separate variables). S4 Table. Factors associated with viral rebound at follow-up among 21,448 CLHIV who had undetectable viral load at baseline in Tanzania (ACHIEVE project interventions reduced into a single binary variable). S5 Table. Factors associated with undetectable viral load at follow-up among 4,809 CLHIV who had detectable viral load at baseline in Tanzania (ACHIEVE project interventions reduced into a single binary variable). (ZIP) [file pone.0349141.s001.zip › Supporting information/S1 Table 5.docx]

| **S1 Table 5. Factors associated with undetectable viral load at follow-up among 4,809 CLHIV who had detectable viral load at baseline in Tanzania (ACHIEVE project interventions reduced into a single binary variable)** | | | | |
| --- | --- | --- | --- | --- |
|  | **adjusted Odds Ratio (aOR)** | **Lower 95% confidence limit** | **Upper 95% confidence limit** | ***p*-value** |
| **ART regimen type** |  |  |  |  |
| DTG-based | 1.000 | — | — | — |
| Other regimens | 0.927 | 0.686 | 1.253 | 0.62 |
| **Months project** |  |  |  |  |
| <6 months | 1.000 | — | — | — |
| 6-11 months | 1.745 | 0.582 | 5.231 | 0.32 |
| 12-23 months | 1.508 | 0.512 | 4.440 | 0.46 |
| **Number of ACHIEVE project interventions received** |  |  |  |  |
| None (0) | 1.000 | — | — | — |
| One or more (≥1) | 1.319 | 1.059 | 1.643 | 0.014 |
| **CLHIV sex** |  |  |  |  |
| Female | 1.000 | — | — | — |
| Male | 0.952 | 0.840 | 1.080 | 0.45 |
| **CLHIV age** |  |  |  |  |
| <5 years | 1.000 | — | — | — |
| 5-9 years | 1.165 | 0.889 | 1.527 | 0.27 |
| 10-14 years | 1.143 | 0.842 | 1.552 | 0.39 |
| 15-17 years | 0.989 | 0.724 | 1.353 | 0.95 |
| **Caregiver age** |  |  |  |  |
| 18-29 years | 1.000 | — | — | — |
| 30-39 years | 1.016 | 0.853 | 1.210 | 0.86 |
| 40-49 years | 1.207 | 1.019 | 1.431 | 0.029 |
| 50-59 years | 1.562 | 1.212 | 2.011 | 0.001 |
| 60+ years | 1.187 | 0.888 | 1.588 | 0.25 |
| **CLHIV school attendance status** |  |  |  |  |
| Not attending | 1.000 | — | — | — |
| Attending school | 0.815 | 0.655 | 1.014 | 0.066 |
| **Level of household hunger** |  |  |  |  |
| Little to no hunger | 1.000 | — | — | — |
| Moderate hunger | 1.065 | 0.889 | 1.276 | 0.49 |
| Severe hunger | 1.160 | 0.799 | 1.683 | 0.44 |
| **Place of residence** |  |  |  |  |
| Rural | 1.000 | — | — | — |
| Urban | 0.803 | 0.699 | 0.922 | 0.002 |
| **Family size** |  |  |  |  |
| 2-3 people | 1.000 | — | — | — |
| 4-6 people | 0.996 | 0.865 | 1.148 | 0.96 |
| 7+ people | 1.089 | 0.653 | 1.815 | 0.74 |
| **Caregiver sex** |  |  |  |  |
| Female | 1.000 | — | — | — |
| Male | 0.975 | 0.853 | 1.115 | 0.71 |
| **Caregiver education** |  |  |  |  |
| Never attended | 1.000 | — | — | — |
| Primary | 0.925 | 0.766 | 1.118 | 0.42 |
| Secondary+ | 1.076 | 0.752 | 1.541 | 0.69 |
| **ART change in the last 6 months** |  |  |  |  |
| No | 1.000 | — | — | — |
| Yes | 0.953 | 0.796 | 1.142 | 0.604 |
| Constant | 1.392 | 0.450 | 4.309 | 0.57 |
