# Supplementary material for: Evaluating the effects of community-based programs on viral rebound and viral suppression among HIV-positive orphaned and vulnerable children receiving antiretroviral treatment: Findings from the ACHIEVE project in Tanzania
Source: PLoS One. 2026 May 15;21(5):e0349141. doi: 10.1371/journal.pone.0349141 (PMC13178892; doi:10.1371/journal.pone.0349141)
Supplement: Attachment — Submitted filename: Response to reviewers R1.pdf [file pone.0349141.s003.pdf]

Response to reviewers' comments

Manuscript title: **Evaluating the effects of community-based programs on viral rebound and viral suppression among HIV-positive orphaned and vulnerable children receiving antiretroviral treatment: findings from the ACHIEVE project in Tanzania**

ID**: PONE-D-25-21420**

Comment	Response
Journal Requirements:<br><br>When submitting your revision, we need you to address these additional requirements.	Overall, we are pleased to submit the revised manuscript after addressing the reviewers' comments. Below, we outline how each comment was addressed.
1. Please ensure that your manuscript meets PLOS ONE's style requirements, including those for file naming. The PLOS ONE style templates can be found at https://journals.plos.org/plosone/s/file?id=wjVg/PLOSOne_formatting_sample_main_body.pdf and https://journals.plos.org/plosone/s/file?id=ba62/PLOSOne_formatting_sample_title_authors_affiliations.pdf	1.The manuscript was revised to ensure that it meets PLOS ONE's style requirements, including those for file naming.
2. We note that the grant information you provided in the 'Funding Information' and 'Financial Disclosure' sections do not match.<br><br>When you resubmit, please ensure that you provide the correct grant numbers for the awards you received for your study in the 'Funding Information' section.	2. We have corrected the statement to ensure that it is the same in both cases. The correct statement is: "*This study draws on data from the ACHIEVE project, which was implemented by Pact in Tanzania with funding from the U.S. President's Emergency Plan for AIDS Relief (PEPFAR) through the former United States Agency for International Development (USAID) under grant number 7200AA19CA00006, awarded to Pact Inc. Authors LK, GS, AE, DK, AK, JC, NM, EK, AB, RF, and TV received salaries through their employment with Pact during the implementation of the ACHIEVE project. The funders had no role in study design, data collection and analysis, decision to publish, or preparation of the manuscript.*"
3. Thank you for stating the following financial disclosure:<br>This paper is based on data from the ACHIEVE project in Tanzania, 2020 - 2026. The project is supported by PEPFAR through the USAID.<br><br>At this time, please address the following queries:	3. We have clarified the following:

a) Please clarify the sources of funding (financial or material support) for your study. List the grants or organizations that supported your study, including funding received from your institution.<br><br>b) State what role the funders took in the study. If the funders had no role in your study, please state: "The funders had no role in study design, data collection and analysis, decision to publish, or preparation of the manuscript."<br><br>c) If any authors received a salary from any of your funders, please state which authors and which funders.<br><br>d) If you did not receive any funding for this study, please state: "The authors received no specific funding for this work."<br><br>Please include your amended statements within your cover letter; we will change the online submission form on your behalf.	(a) The only source of funding was PEPFAR, and this has been clarified.<br><br>(b) As guided, we have also added the statement that "The funders had no role in study design, data collection and analysis, decision to publish, or preparation of the manuscript".<br><br>(c) We have also stated the authors who received salaries through their employment with Pact during the implementation of the ACHIEVE project.<br><br>(d) Not applicable
4. Thank you for stating the following in the Acknowledgments Section of your manuscript: This study is part of the ACHIEVE project, funded by PEPFAR through USAID. A version of this manuscript, based on Group 1 respondents, was presented as a poster (WEPEC296) at AIDS 2024, the 25th International AIDS Conference, held in Munich, Germany, from July 22-26, 2024. Further details about the presentation can be found at: https://programme.aids2024.org/Abstract/Abstract/?abstractid=3356. Also, another abstract based on Group 2 respondents was presented as a poster (TUPE040) at the HIVR4P 2024, the 5th HIV Research for Prevention Conference that took place in Lima, Peru, October 6-10, 2024.<br><br>We note that you have provided funding information that is not currently declared in your Funding Statement. However, funding information should not appear in the Acknowledgments section or other areas of your manuscript. We will only publish funding information present in the Funding Statement section of the online submission form. Please remove any funding-related text from the manuscript and let us know how you would like to update your Funding Statement. Currently, your Funding Statement reads as follows:	4. We have amended the funding statement accordingly and included it in the cover letter as guided. We have also removed any funding-related text from the manuscript.

This paper is based on data from the ACHIEVE project in Tanzania, 2020 - 2026. The project is supported by PEPFAR through the USAID.<br><br>Please include your amended statements within your cover letter; we will change the online submission form on your behalf.	
5. Thank you for stating the following in the Competing Interests section:<br>I have read the journal's policy and the authors of this manuscript have the following competing interests: One author, Amon Exavery, was awarded a travel scholarship by the International AIDS Society (IAS) to attend and present an abstract based on Group 2 respondents at the HIVR4P 2024, the 5th HIV Research for Prevention Conference, held in Lima, Peru, October 6–10, 2024. The rest of the authors declared that no competing interests exist.<br><br>Please confirm that this does not alter your adherence to all PLOS ONE policies on sharing data and materials, by including the following statement: "This does not alter our adherence to PLOS ONE policies on sharing data and materials." (as detailed online in our guide for authors http://journals.plos.org/plosone/s/competing-interests). If there are restrictions on sharing of data and/or materials, please state these. Please note that we cannot proceed with consideration of your article until this information has been declared.<br>Please include your updated Competing Interests statement in your cover letter; we will change the online submission form on your behalf.	5. As advised, we have updated the Conflicts of Interests statement, and now reads "*One author, Amon Exavery, was awarded a travel scholarship by the International AIDS Society (IAS) to attend and present an abstract based on Group 2 respondents at HIVR4P 2024, the 5th HIV Research for Prevention Conference, held in Lima, Peru, October 6–10, 2024. The rest of the authors declare that they do not have any competing interests. This does not alter our adherence to PLOS ONE policies on sharing data and materials.*" This statement is included in the cover letter, and we have removed what we had stated earlier in the manuscript.
6. In the online submission form, you indicated that deidentified participant data will be available upon reasonable request. Any additional requests for data access or analysis must receive prior approval from Pact. Such requests should be directed to Dr. Levina Kikoyo at lkikoyo@pactworld.org.<br><br>All PLOS journals now require all data underlying the findings described in their manuscript to be freely available to other researchers, either a. In a public repository, b. Within the manuscript itself, or c. Uploaded as supplementary information.<br>This policy applies to all data except where public deposition would breach compliance with the	(6) We have revised the text to indicate that all relevant data are within the manuscript and its Supporting information. Thank you.

protocol approved by your research ethics board. If your data cannot be made publicly available for ethical or legal reasons (e.g., public availability would compromise patient privacy), please explain your reasons on resubmission and your exemption request will be escalated for approval.	
7. If the reviewer comments include a recommendation to cite specific previously published works, please review and evaluate these publications to determine whether they are relevant and should be cited. There is no requirement to cite these works unless the editor has indicated otherwise.	(7) Thank you for the guidance. We carefully reviewed the publications suggested by the reviewer, assessed their relevance to the study, and only cited those that we considered appropriate and directly relevant to the manuscript.
**Additional Editor Comments:**	**Responses to Additional Editor Comments:**
The manuscript needs serious attention to eliminate inconsistencies and improve clarity. Some of the inconsistencies have been presented under comments from reviewer 1.<br><br>In addition, kind attend to the following:	
1. Reconcile names of community program in the your letter to the editor (currently stated as 'WORTH Yetu economic strengthening') with the name presented in your manuscript title (ACHIEVE).	1. We apologize for the misunderstanding. We have clarified in both the letter to the editor and the revised manuscript that ACHIEVE is the overall project name, from which three specific interventions, (1) WORTH Yetu, (2) linkage to teen/paediatric clubs, and (3) health insurance, were evaluated in the present study for their associations with two clinical outcomes, (1) viral rebound and (2) achieving an undetectable viral load among HIV-positive orphaned and vulnerable children aged 0-17 years receiving antiretroviral therapy (ART) in Tanzania.
2. Your study title and abstract seem to represent just a subset of the entire study. The first two study objectives presented in the main body (lines 111-118) are not captured in your title and abstract. Moreover, the evaluation of 'the impact of ACHIEVE' as an objective is so marginalized that a reader would consider it a secondary objective. I would encourage authors to rethink their study report and line it with their original study aims.	2. We revised the title to reflect the assessment of two outcomes: viral rebound and viral suppression (Lines 4–7). In addition, we clarified in the Methods section of the abstract that this was achieved using two analytic groups: Group 1 (viral rebound, n = 21,448) and Group 2 (undetectable viral load, n = 4,809) (Lines 35–45).<br><br>We removed the objective related to the impact of ACHIEVE to better align the study report with the original study aims and maintain a clear and focused presentation of the primary objectives.

3. Just to emphasize a comment by reviewer 1, of the need for authors to clearly state the inclusion/ exclusion criteria, the report seems to mix up 'period on ART' and 'duration under ACHIEVE'. While lines 158-160 states that CLHIV who had been on ART for at least six months and had at least two clinically confirmed viral load test results spaced at least six months apart between July 2021 and July 2023', the table of baseline characteristics on page 12 emphasizes on duration on the ACHIEVE project', and goes ahead to include participants' duration less than 6 months. A reader would wonder if this group was eligible for the study. The authors therefore need to clearly state the inclusion/ exclusion criteria, and consistently apply the relevant variables in the analysis.	3. Inclusion criteria are clarified, lines 154–156. Duration on ART is a clinical characteristic that informs the appropriate timing for viral load testing according to the national guidelines. In this study, we did not analyze duration on ART; it was mentioned only to explain the viral load data and how it was used in the present study. The variable we analyzed was duration in the ACHIEVE project, which is not the same as duration on ART. For example, some children were enrolled in the ACHIEVE project while already receiving ART. Therefore, these children had longer ART exposure but shorter time in ACHIEVE, which explains why, for instance, the 44 children in Table 2 had been in the ACHIEVE project for less than six months but had been on ART for six or more months.
4. The tables of results are overcrowded with a lot information. Authors should consider limiting themselves to data most relevant to their report. Just to cite examples: Is it of much relevant to your report that caregiver's age be categorized into 5 groups and then family size into 3 groups?	4. We carefully considered the suggestion to reduce the amount of information presented in the tables and made efforts to avoid including unnecessary details (note that one reviewer had suggested adding data). As part of this, we removed confidence in intervals in Table 3 to make lighter. However, we retained selected categorical variables, such as caregiver age and family size, to support transparency and analytical completeness, as these categorizations are consistent with approaches used in the related literature (e.g., https://doi.org/10.1371/journal.pone.0264315 and https://doi.org/10.1371/journal.pone.0301578). While the tables present detailed results for readers who may wish to explore the data further, the manuscript text focuses on the most relevant findings to preserve clarity and readability. This approach was intended to balance conciseness with transparency in reporting the results
Reviewers' comments:<br><br>Reviewer's Responses to Questions<br>**Comments to the Author**<br><br>1. Is the manuscript technically sound, and do the data support the conclusions?<br><br>The manuscript must describe a technically sound	**Responses to Reviewers' comments:**

piece of scientific research with data that supports the conclusions. Experiments must have been conducted rigorously, with appropriate controls, replication, and sample sizes. The conclusions must be drawn appropriately based on the data presented.<br>Reviewer #1: Yes<br>Reviewer #2: Yes	 <br><br><br><br><br>Thank you.<br>Thank you.
2. Has the statistical analysis been performed appropriately and rigorously?<br>Reviewer #1: Yes<br>Reviewer #2: Yes	 <br>Thank you.<br>Thank you.
3. Have the authors made all data underlying the findings in their manuscript fully available?<br><br>The PLOS Data policy requires authors to make all data underlying the findings described in their manuscript fully available without restriction, with rare exception (please refer to the Data Availability Statement in the manuscript PDF file). The data should be provided as part of the manuscript or its supporting information, or deposited to a public repository. For example, in addition to summary statistics, the data points behind means, medians and variance measures should be available. If there are restrictions on publicly sharing data—e.g. participant privacy or use of data from a third party—those must be specified.<br>Reviewer #1: Yes<br>Reviewer #2: Yes	Thank you.<br>Thank you.
4. Is the manuscript presented in an intelligible fashion and written in standard English?<br><br>PLOS ONE does not copyedit accepted manuscripts, so the language in submitted articles must be clear, correct, and unambiguous. Any typographical or grammatical errors should be corrected at revision, so please note any specific errors here.<br>Reviewer #1: Yes<br>Reviewer #2: Yes	Thank you.<br>Thank you.
5. Review Comments to the Author<br><br>Please use the space provided to explain your answers to the questions above. You may also include additional comments for the author, including concerns about dual publication, research	

ethics, or publication ethics. (Please upload your review as an attachment if it exceeds 20,000 characters)	
Reviewer #1: This manuscript is technically sound and result of their findings supports the conclusion. The presented data, the methodology and the details of the analysis shows that they used standard data analysis software and were able to different variables and possible factors that could affect the outcome. However, I observe that the authors needs few major revisions on some of the Tables as I have highlighted in the manuscript to aid the readers to appreaciate the result of their findings in a more simplified and clear fashion without much ambiguity.<br><br>I have made comments on the body of the work as they would find in the attached document (*pasted below by the authors*).	Thank you for the encouraging feedback on the manuscript. We have carefully reviewed all the comments and outline below how each has been addressed:
1. line 83, put a period "after [3]"	1. Corrected, thank you. Line 83.
2. line 146 - Enrollment criteria should be clearly stated.	2. Enrollment criteria have been stated, Lines 154–156.
3. Line 223 to 227: Just wondering why the household hunger was well explained and detailed with the categorizations but other variables were not. Try and stay consistent.	3. We provided a more detailed explanation for household hunger because it is a derived variable with specific categorizations that required clarification for transparency and interpretability. In contrast, other variables such as age, sex, and education are standard demographic measures that are widely understood and therefore did not require a similar level of elaboration. So, we believe that the current description of the variables in the manuscript is sufficient and clear.
4. Line 222 to 224: Caregiver marital status, and disability status were listed but were not found in Table 2. Also, be consistent by prefix caregivers variables as appropriate. For example, Caregiver Martial status rather than Marital status., Caregiver Place of Residence etc.	4. Thank you for this observation. Caregiver marital status and caregiver disability status were initially considered for inclusion; however, they were dropped during analysis due to technical considerations. To ensure consistency between the text and Table 2, these variables have been removed from the list. In addition, all caregiver-level variables have now been consistently prefixed as “Caregiver” throughout the manuscript, as suggested.
5. Line 222" In table 2, you have CLHIV school attendance status but here you have	5. Thank you for noting this inconsistency. We have corrected the text to ensure consistency

school enrollment status. Try to be consistent. Stick with one either enrollment or attendance because they could mean different things.	with Table 2 and have used *school attendance* throughout the manuscript.
6. Line 227: There is a bit of inconsistency here. Just wondering why household hunger was singled out and was well explained and categorized but not with the same level of details for other independent variable.	6. As described in the response to comment 3 above, we provided a more detailed explanation for household hunger because it is a derived variable with specific categorizations that required clarification for transparency and interpretability. In contrast, other variables such as age, sex, and education are standard demographic measures that are widely understood and therefore did not require a similar level of elaboration. So, we believe that the current description of the variables in the manuscript is sufficient and clear.
7. Line 267: this statement "and the rest were on other regimens (2.8%)." is no longer relevant.	7. Thank you for pointing this out. The unnecessary statement has been deleted from the manuscript, Line 300.
8. Line 153: S1 as seen in the documents identifying the tables are not clear as one could not find corresponding S1 tables in the document. There is need for consistency. I assume that the table under supporting documents will be placed in their proper place to take care of this concern.	8. In line 151 (now Lines 167-168), the referenced table "S1 Table 1" was included as part of the Supporting Information, contained in a zipped folder. The link to this folder is provided on the last page of the PDF manuscript file you reviewed.
9. Line 314: could not find the value for WORTH Yetu as referenced in the table 3. Be sure that the results described are properly referenced in the Table.	9. Thank you for this comment. The figures for WORTH Yetu, as well as the other interventions referenced, are presented in Table 3. Specifically, viral rebound among CLHIV whose caregivers participated in WORTH Yetu was 12.5% compared to 13.2% among those who did not participate; the difference was not statistically significant (p = 0.250). To improve clarity and ensure accurate cross-referencing between the text and Table 3, we have revised the sentence in Lines 347–348 to explicitly align the narrative with the values reported in the table.
10. Table 3: I would suggest column total for this table to enable the readers to appreciate the comparison using the population for each sub-group.	10. In Table 3, percentages are presented within each subgroup, with viral rebound (detectable viral load at follow-up) treated as the outcome category of interest. For

The results are a bit confusing and difficult to interpret.	example, viral rebound occurred in 12.9% of CLHIV on DTG-based regimens compared with 17.8% among those on other regimens. This row-wise presentation allows direct comparison of rebound across levels of each covariate. The corresponding subgroup sizes for all covariates are provided in the frequency distribution table (Table 2), which serves as the denominator reference. This approach was applied consistently across all variables for both Group 1 and Group 2 outcomes to avoid redundancy while maintaining clarity. Thank you.
11. Table 4: "Number of ACHIEVE Project intervention received". The categories should be numbers, Either you go with Zero, One, Two and Three or 0, 1, 2,& 3. There is no need for the numbers in the bracket.	11. Thank you for this comment. The requested changes to the "Number of ACHIEVE Project interventions received" categories have been applied in both Tables 4, 5 & 6 and the corresponding text. The categories now use text labels ("Zero," "One," "Two," and "Three") without brackets, and all references have been aligned with Table 4 for consistency.
12. Line 275: under the Duration in the Achieve project, you have 3 categories. The question is what is the relevance of categorizing <6months that has only 44 CLHIV which was used as a reference point in Table 4&5 hence diluting the result of other 2 categorizes because of its wide gap as compared with the other categories. For example in Table 5, those on ACHIEVE project for 6 months and more are more likely to be undetectable as shown in the table though not significant between 6-11months and 12-23 months. In real life, one would have expected those longer in the program to have more likelihood of having undetectable viral load than those that are 6-11months. Hence my concern for the categorization. It will be nice to see the result for just 2 categories, say less than 1 year and 1 year to 2years.	12. Thank you for this insightful comment. The category <6 months was intentionally included to capture children who were newly enrolled in the ACHIEVE project (i.e., had been in the project for two consecutive quarters) compared with those who had been in the project longer. Although this category included a smaller number of children (n = 44) compared with the other groups, the sample size was still sufficient for the statistical analyses conducted. As advised, we also conducted a separate analysis recategorizing duration as <1 year versus ≥1 year, and the results remained largely unchanged, with the variable still not statistically significant. Therefore, we retained the original three-category classification to reflect programmatic distinctions while maintaining statistical validity.
13. Line 340" This is not specific. Regression analysis is broad. Please be specific to logistic regression.	13. Thank you for this comment. We have added the missing word – logistic – to make it specific, Lines 375, 376, 379.

Reviewer #2: Thank you for the opportunity to review this interesting article on community base programming and the influence of three categories of interventions on viral load among children receiving ARVs.<br><br>While the article is potentially of interest to a subset of readers who are programming with certain pediatric populations in mind, the interventions that were the focus of this report are not especially innovative, and the specifics of these interventions is unclear. Even if one is to pursue similar programming based on these findings, it would be nearly impossible to reproduce the activities leading to the reported impact.<br><br>Importantly, there is very little discussion of sustainability, cost-effectiveness, government ownership and the current context of HIV programming (including community-level investments) as of this review in late 2025.<br><br>In fact, for those following the future of programming and related budgets in Tanzania and other countries in the region, the manuscript comes off as disjointed from reality and largely naive to the latest driving programmatic political factors that will ultimately determine the success of such programs, and their relevance.<br><br>Indeed, the funding agency that is behind these investments no longer exists, and the upcoming five years will look nothing like the previous 20. If one is to look for similar studies over the last decade, s/he will find several, including from Tanzania.*** While the Konga community-based intervention model seems to be implemented by the National Council of People Living with HIV (NACOPHA), the relationship of current work with he many years preceding would offer important context. To imporve the value of this manuscript. the report needs to include much more information placing this study in current context.<br><br>With a re-write to update the paper in accordance with current context and placing these interventions in context (and providing more information on how the activities are executed so that they can be replicated), the Editor may consider accepting the article. Of note, much of the	We are thankful to the reviewer for the detailed and constructive feedback. This study provides an evidence-based contribution by evaluating real-world community-based interventions within the Tanzanian context, generating evidence of their associations with viral load outcomes among orphaned and vulnerable children living with HIV (CLHIV). Although the interventions examined may not be novel in isolation, the contribution of this work lies in rigorously assessing these interventions in a non-experimental setting aligned with the design of the ACHIEVE project. To address the concern regarding intervention detail and reproducibility, we have provided additional descriptions of the three interventions – WORTH Yetu, linkage to teen/paediatric clubs, and health insurance – and clarified the role of the ACHIEVE project in each (Lines 221–248).<br><br>Furthermore, most comparable studies in the literature (including those suggested by the reviewer) are randomized by design, which was not feasible given the implementation structure of the ACHIEVE project. Evaluating non-experimental community-based interventions presents well-recognized methodological challenges; this study addresses these through appropriate longitudinal and multilevel analytical approaches, contributing valuable evidence in a context where data among CLHIV remain limited.<br><br>With respect to sustainability, cost-effectiveness, and government ownership, these were not primary objectives of the current analysis, and no empirical results were generated to support a formal discussion of these domains. We have therefore revised the discussion to note that these remain important areas for future research, particularly in light of current donor funding uncertainties. The revised text highlights the need for government-led mechanisms to fully integrate and maintain effective community-level interventions within existing health systems, ensuring that the benefits are sustained for the well-being of CLHIV and other PLHIV (Lines 504–520).

existing literature focuses on adolescents and youth, and CLHIV data is sparse.<br><br>A rooted paper informing the future of practical community programming among this young and highly vulnerable population would be welcome.<br><br>---<br>***<br>Ferrand RA et al. 2017. Community-based caregiver support to reduce virological failure in children and adolescents with HIV (ZENITH): Randomised controlled trial. Lancet Child Adolesc Health.<br><br>Chikwari CD et al. 2018. Community health worker support for older children and adolescents with HIV: Process evaluation of the ZENITH trial. Implementation Science.<br><br>Willis N et al. 2019. Effectiveness of Community Adolescent Treatment Supporters (CATS) on linkage, retention, and adherence among adolescents with HIV: Randomised trial. BMC Public Health.<br><br>Mavhu W et al. 2020. Differentiated peer-led service delivery for adolescents with HIV (Zvandiri): Cluster randomised trial. Lancet Global Health.<br><br>Ndhlovu CE et al. 2021. Peer-support intervention to improve ART outcomes among adolescents and young adults with virologic failure: Randomised controlled trial. AIDS Research & Therapy.<br><br>Simms V et al. 2022. Peer-led Problem Discussion Therapy for adolescents living with HIV: Cluster-randomised trial. PLOS Medicine.<br><br>Dhlamini N et al. 2019. Peer-led HIV care continuum outcomes in the Zvandiri programme. Global Health: Science & Practice.<br><br>Mageda K et al. 2023. Effectiveness of a community-based Konga model to improve viral suppression in children with HIV: Cluster-randomised clinical trial. BMC Public Health.<br><br>Mongi J et al. 2023. Community-based differentiated service delivery for paediatric HIV in	To situate this work within the broader HIV programming context, the introduction and discussion sections now provide additional contextual information on community-level investments and the Konga community-based intervention model implemented by NACOPHA. This positions the study as a practical, evidence-informed contribution to child-focused community programming for a highly vulnerable population, addressing gaps in empirical data for CLHIV in Tanzania (Lines 95–124 and 504–520).

Tanzania: Improved viral suppression and retention. HIV Pediatrics Conference Proceedings.<br><br>Msomi N et al. 2023. Determinants of viral load suppression among orphaned and vulnerable children receiving community-based caseworker support. Frontiers in Public Health.<br><br>Kahema L et al. 2022. Determinants of viral load non-suppression among HIV-positive children and adolescents in community treatment settings in Tanzania. Bulletin of the National Research Centre.<br><br>Grimwood A et al. 2012. Community adherence support and treatment outcomes among children on ART: Multicentre cohort study. Journal of the International AIDS Society.<br><br>Machiha A et al. 2024/2025. Integrated community-based HIV and SRH services for youth (CHIEDZA): Cluster randomised trial. Nature Medicine.	
6. PLOS authors have the option to publish the peer review history of their article (what does this mean?). If published, this will include your full peer review and any attached files.<br><br>If you choose "no", your identity will remain anonymous but your review may still be made public.<br><br>**Do you want your identity to be public for this peer review?** For information about this choice, including consent withdrawal, please see our Privacy Policy.<br>Reviewer #1: **Yes:** Nnenna Assumpta EZEOKAFOR<br>Reviewer #2: No	Thank you.

END
