# Supplementary material for: Evaluating the effects of community-based programs on viral rebound and viral suppression among HIV-positive orphaned and vulnerable children receiving antiretroviral treatment: Findings from the ACHIEVE project in Tanzania
Source: PLoS One. 2026 May 15;21(5):e0349141. doi: 10.1371/journal.pone.0349141 (PMC13178892; doi:10.1371/journal.pone.0349141)
Supplement: Attachment — Submitted filename: PONE-D-25-21420R1.pdf [file pone.0349141.s004.pdf]

PONE-D-25-21420R1: Reviewer Comment for Title

1. Pdf Page 9_Line 5: Use of HIV-positive: this terminology is not appropriate per UNAIDS guidance. Replace with “living with HIV”
