# Supplementary material for: Evaluating the effects of community-based programs on viral rebound and viral suppression among HIV-positive orphaned and vulnerable children receiving antiretroviral treatment: Findings from the ACHIEVE project in Tanzania
Source: PLoS One. 2026 May 15;21(5):e0349141. doi: 10.1371/journal.pone.0349141 (PMC13178892; doi:10.1371/journal.pone.0349141)
Supplement: Attachment — Submitted filename: Response to Reviewers.docx [file pone.0349141.s006.docx]

**Response to reviewers’ comments**

Manuscript title: **Evaluating the effects of community-based programs on viral rebound and viral suppression among HIV-positive orphaned and vulnerable children receiving antiretroviral treatment: findings from the ACHIEVE project in Tanzania**

ID**: PONE-D-25-21420R1**

| **Comment** | **Response** |
| --- | --- |
| PONE-D-25-21420R1  Evaluating the effects of community-based programs on viral rebound and viral suppression among HIV-positive orphaned and vulnerable children receiving antiretroviral treatment: findings from the ACHIEVE project in Tanzania  PLOS One  Dear Dr. Exavery,  Thank you for submitting your manuscript to PLOS ONE. After careful consideration, we feel that it has merit but does not fully meet PLOS ONE’s publication criteria as it currently stands. Therefore, we invite you to submit a revised version of the manuscript that addresses the points raised during the review process.  Please submit your revised manuscript by May 10 2026 11:59PM. If you will need more time than this to complete your revisions, please reply to this message or contact the journal office at [plosone@plos.org](mailto:plosone@plos.org). When you're ready to submit your revision, log on to <https://www.editorialmanager.com/pone/> and select the 'Submissions Needing Revision' folder to locate your manuscript file.  Please include the following items when submitting your revised manuscript:   - A letter that responds to each point raised by the academic editor and reviewer(s). You should upload this letter as a separate file labeled 'Response to Reviewers'. - A marked-up copy of your manuscript that highlights changes made to the original version. You should upload this as a separate file labeled 'Revised Manuscript with Track Changes'. - An unmarked version of your revised paper without tracked changes. You should upload this as a separate file labeled 'Manuscript'.   If you would like to make changes to your financial disclosure, please include your updated statement in your cover letter. Guidelines for resubmitting your figure files are available below the reviewer comments at the end of this letter.  If applicable, we recommend that you deposit your laboratory protocols in protocols.io to enhance the reproducibility of your results. Protocols.io assigns your protocol its own identifier (DOI) so that it can be cited independently in the future. For instructions see: [https://journals.plos.org/plosone/s/submission-guidelines#loc-laboratory-protocols](https://nam11.safelinks.protection.outlook.com/?url=https%3A%2F%2Ftrack.editorialmanager.com%2FCL0%2Fhttps%3A%252F%252Fjournals.plos.org%252Fplosone%252Fs%252Fsubmission-guidelines%2523loc-laboratory-protocols%2F1%2F010f019d2cf426c9-7090cacc-61cb-4015-a300-1e2b85f14027-000000%2F3spTShNrUiSYYumvNCqphqgCf6bPqNvVx3leBA-aUWk%3D256&data=05%7C02%7Caexavery%40pactworld.org%7C1cac0ef03aa241b79e6e08de8ba1f98c%7C3973ea966d9046bf860543454d6905fc%7C0%7C0%7C639101725557483861%7CUnknown%7CTWFpbGZsb3d8eyJFbXB0eU1hcGkiOnRydWUsIlYiOiIwLjAuMDAwMCIsIlAiOiJXaW4zMiIsIkFOIjoiTWFpbCIsIldUIjoyfQ%3D%3D%7C0%7C%7C%7C&sdata=FDmKXokXy8wBC3H9QcalwBlTSwObImebPz%2B%2BD0578j4%3D&reserved=0). Additionally, PLOS ONE offers an option for publishing peer-reviewed Lab Protocol articles, which describe protocols hosted on protocols.io. Read more information on sharing protocols at [https://plos.org/protocols?utm_medium=editorial-email&utm_source=authorletters&utm_campaign=protocols](https://nam11.safelinks.protection.outlook.com/?url=https%3A%2F%2Ftrack.editorialmanager.com%2FCL0%2Fhttps%3A%252F%252Fplos.org%252Fprotocols%253Futm_medium%3Deditorial-email%2526utm_source%3Dauthorletters%2526utm_campaign%3Dprotocols%2F1%2F010f019d2cf426c9-7090cacc-61cb-4015-a300-1e2b85f14027-000000%2FJsda1OSNSUj_2Fqj7qzSua_1XfF8Bg58HQLzDmFN0ZI%3D256&data=05%7C02%7Caexavery%40pactworld.org%7C1cac0ef03aa241b79e6e08de8ba1f98c%7C3973ea966d9046bf860543454d6905fc%7C0%7C0%7C639101725557509417%7CUnknown%7CTWFpbGZsb3d8eyJFbXB0eU1hcGkiOnRydWUsIlYiOiIwLjAuMDAwMCIsIlAiOiJXaW4zMiIsIkFOIjoiTWFpbCIsIldUIjoyfQ%3D%3D%7C0%7C%7C%7C&sdata=0M%2F07v39FMGnGVcOxW%2FTOypgryXM19WcXlLc1F2v3kI%3D&reserved=0).  As the corresponding author, your ORCID iD is verified in the submission system and will appear in the published article. PLOS supports the use of ORCID, and we encourage all coauthors to register for an ORCID iD and use it as well. Please encourage your coauthors to verify their ORCID iD within the submission system before final acceptance, as unverified ORCID iDs will not appear in the published article. *Only* the individual author can complete the verification step; PLOS staff *cannot* verify ORCID iDs on behalf of authors.  We look forward to receiving your revised manuscript.  Kind regards,  Tacilta Nhampossa Academic Editor PLOS One  **Journal Requirements:**  1, If the reviewer comments include a recommendation to cite specific previously published works, please review and evaluate these publications to determine whether they are relevant and should be cited. There is no requirement to cite these works unless the editor has indicated otherwise.   2. Please review your reference list to ensure that it is complete and correct. If you have cited papers that have been retracted, please include the rationale for doing so in the manuscript text, or remove these references and replace them with relevant current references. Any changes to the reference list should be mentioned in the rebuttal letter that accompanies your revised manuscript. If you need to cite a retracted article, indicate the article’s retracted status in the References list and also include a citation and full reference for the retraction notice.  [Note: HTML markup is below. Please do not edit.]  Reviewers' comments:  Reviewer's Responses to Questions  **Comments to the Author**  1. If the authors have adequately addressed your comments raised in a previous round of review and you feel that this manuscript is now acceptable for publication, you may indicate that here to bypass the “Comments to the Author” section, enter your conflict of interest statement in the “Confidential to Editor” section, and submit your "Accept" recommendation.  Reviewer #1: All comments have been addressed  Reviewer #2: All comments have been addressed  Reviewer #3: All comments have been addressed  2. Is the manuscript technically sound, and do the data support the conclusions?  The manuscript must describe a technically sound piece of scientific research with data that supports the conclusions. Experiments must have been conducted rigorously, with appropriate controls, replication, and sample sizes. The conclusions must be drawn appropriately based on the data presented.  Reviewer #1: Yes  Reviewer #2: Yes  Reviewer #3: Yes  3. Has the statistical analysis been performed appropriately and rigorously?  Reviewer #1: Yes  Reviewer #2: Yes  Reviewer #3: Yes  4. Have the authors made all data underlying the findings in their manuscript fully available?  The [PLOS Data policy](https://nam11.safelinks.protection.outlook.com/?url=http%3A%2F%2Ftrack.editorialmanager.com%2FCL0%2Fhttp%3A%252F%252Fwww.plosone.org%252Fstatic%252Fpolicies.action%2523sharing%2F1%2F010f019d2cf426c9-7090cacc-61cb-4015-a300-1e2b85f14027-000000%2FRogrzlYVAXEuyME9SJ5DAkoOHDtRJf8t7mb1h6IH8bY%3D256&data=05%7C02%7Caexavery%40pactworld.org%7C1cac0ef03aa241b79e6e08de8ba1f98c%7C3973ea966d9046bf860543454d6905fc%7C0%7C0%7C639101725557530827%7CUnknown%7CTWFpbGZsb3d8eyJFbXB0eU1hcGkiOnRydWUsIlYiOiIwLjAuMDAwMCIsIlAiOiJXaW4zMiIsIkFOIjoiTWFpbCIsIldUIjoyfQ%3D%3D%7C0%7C%7C%7C&sdata=o7LVhDOZkp45g8eDN3G4jU8DbDMt5HHZ2A0oKll5OIg%3D&reserved=0) requires authors to make all data underlying the findings described in their manuscript fully available without restriction, with rare exception (please refer to the Data Availability Statement in the manuscript PDF file). The data should be provided as part of the manuscript or its supporting information, or deposited to a public repository. For example, in addition to summary statistics, the data points behind means, medians and variance measures should be available. If there are restrictions on publicly sharing data—e.g. participant privacy or use of data from a third party—those must be specified.  Reviewer #1: Yes  Reviewer #2: Yes  Reviewer #3: Yes  5. Is the manuscript presented in an intelligible fashion and written in standard English?  PLOS ONE does not copyedit accepted manuscripts, so the language in submitted articles must be clear, correct, and unambiguous. Any typographical or grammatical errors should be corrected at revision, so please note any specific errors here.  Reviewer #1: Yes  Reviewer #2: Yes  Reviewer #3: Yes  6. Review Comments to the Author  Please use the space provided to explain your answers to the questions above. You may also include additional comments for the author, including concerns about dual publication, research ethics, or publication ethics. (Please upload your review as an attachment if it exceeds 20,000 characters)  Reviewer #1: The authors have adequately addressed the comments raised in the previous round of review; however, there are a few minor revisions I ave observed that should be corrected before publication. I have highlighted the comments in the attached document  Reviewer #2: This reviewer appreciates the thoughtful responses to all elements in the initial review. Thank you for the opportunity to provide feedback.  Reviewer #3: One main comment about the title (line 5 of the PDF version: Use of the term "HIV-positive": this terminology is not appropriate per UNAIDS guidance. Replace with “living with HIV”.  7. PLOS authors have the option to publish the peer review history of their article ([what does this mean?](https://nam11.safelinks.protection.outlook.com/?url=https%3A%2F%2Ftrack.editorialmanager.com%2FCL0%2Fhttps%3A%252F%252Fjournals.plos.org%252Fplosone%252Fs%252Feditorial-and-peer-review-process%2523loc-peer-review-history%2F1%2F010f019d2cf426c9-7090cacc-61cb-4015-a300-1e2b85f14027-000000%2Fxd5WhAKQsZUGPHjgsiAWmwBLQhTG9W37_CI8fwKR6Z4%3D256&data=05%7C02%7Caexavery%40pactworld.org%7C1cac0ef03aa241b79e6e08de8ba1f98c%7C3973ea966d9046bf860543454d6905fc%7C0%7C0%7C639101725557551920%7CUnknown%7CTWFpbGZsb3d8eyJFbXB0eU1hcGkiOnRydWUsIlYiOiIwLjAuMDAwMCIsIlAiOiJXaW4zMiIsIkFOIjoiTWFpbCIsIldUIjoyfQ%3D%3D%7C0%7C%7C%7C&sdata=%2BdD5ITsktioeay0pZnAyEnZFD8fuknEiRQduheFCKKc%3D&reserved=0)). If published, this will include your full peer review and any attached files.  If you choose “no”, your identity will remain anonymous but your review may still be made public. **Do you want your identity to be public for this peer review?** For information about this choice, including consent withdrawal, please see our [Privacy Policy](https://nam11.safelinks.protection.outlook.com/?url=https%3A%2F%2Ftrack.editorialmanager.com%2FCL0%2Fhttps%3A%252F%252Fwww.plos.org%252Fprivacy-policy%2F1%2F010f019d2cf426c9-7090cacc-61cb-4015-a300-1e2b85f14027-000000%2F-rTjQie2B2ak1ie9hYbX2Fo44NJ2WHcssyeKQTWVhpc%3D256&data=05%7C02%7Caexavery%40pactworld.org%7C1cac0ef03aa241b79e6e08de8ba1f98c%7C3973ea966d9046bf860543454d6905fc%7C0%7C0%7C639101725557573060%7CUnknown%7CTWFpbGZsb3d8eyJFbXB0eU1hcGkiOnRydWUsIlYiOiIwLjAuMDAwMCIsIlAiOiJXaW4zMiIsIkFOIjoiTWFpbCIsIldUIjoyfQ%3D%3D%7C0%7C%7C%7C&sdata=y9NnD49wSr7TucJ19lyKwT%2BtaRGq5%2Fz%2F4Pib69%2FUjhM%3D&reserved=0).  Reviewer #1: No  Reviewer #2: No  Reviewer #3: No  [NOTE: If reviewer comments were submitted as an attachment file, they will be attached to this email and accessible via the submission site. Please log into your account, locate the manuscript record, and check for the action link "View Attachments". If this link does not appear, there are no attachment files.]    To ensure your figures meet our technical requirements, please review our figure guidelines: <https://journals.plos.org/plosone/s/figures>  You may also use PLOS’s free figure tool, NAAS, to help you prepare publication quality figures: <https://journals.plos.org/plosone/s/figures#loc-tools-for-figure-preparation>.  NAAS will assess whether your figures meet our technical requirements by comparing each figure against our figure specifications.  **Comments from attachments** (*pasted below by the authors*)  *Comments on clean manuscript*   - Pdf Page 9_Line 5: Use of HIV-positive: this terminology is not appropriate per UNAIDS guidance. Replace with “living with HIV” - **Line 201–202**: Not same with   *Comments on manuscript with track changes*   - **Line 27**: This (referring to CLHIV) should not be deleted as the acronym will be reflected later in other parts of the document - **Line 31**: CLHIV- stands for Children Living with HIV, and this is the standard translation of this acronym. it is not the same as HIV-positive orphaned and vulnerable children. OVC covers a wider population that includes HIV-negative children affected by HIV/AIDS. Maybe you can introduce your acronym. eg; HPOVC or something similar or stay with CLHIV but the full meaning needs to reflected as what it is. - **Line 105:** Please use CLHIV that you have already defined rather than introduce another acronym "LHIV". - **Line 133–134**: used the ACHIEVE acronym instead. this is already defined in the introduction. - **Line 206:** I guess you meant that all caregivers & CLHIV were eligible for them. please confirm and update accordingly. - **Line 214–217**: This should not be part of the methodology but should be in the result section, so expunge. I observed it is already reported in the result but with inconsistent figures. For instance 19.2% was reported as 19.3% in line 302. 26.1% here but reported as 26.6% in line 305. 87.5% here but reported as 87.4% in line 304.   Please double check and update accordingly.   - **Table 3**: Not sure why number range was canceled as it provides useful information. - **Line 504**: I would recommend that you use the findings suggest. | We sincerely thank you for giving us another opportunity to refine the manuscript based on the feedback received from the reviewers on 27 March 2026. All comments have been carefully addressed, as outlined below:   - A ‘Response to Reviewers’ file outlining our responses to each point raised by the academic editor and reviewers has been uploaded. - A ‘Revised Manuscript with Track Changes’ file highlighting the changes made to the original version of the manuscript has been uploaded. - A ‘Manuscript’ file constituting an unmarked version of the revised paper without tracked changes has been uploaded.   No changes to the financial disclosure statement.  Not applicable.  Thank you.  1. No recommendation to cite previous works. Thank you.  2. We have reviewed the reference list and confirm that it is complete and accurate.  Response to reviewers’ comments  Thank you.  Thank you.  Thank you.  Thank you.  Thank you.  Thank you.  Thank you.  Thank you.  Thank you.  Thank you.  Thank you.  Thank you.  Thank you.  Thank you.  Thank you.  All comments provided in the attachment have been reviewed and addressed accordingly. Thank you.  Thank you.  We revisited the 2024 UNAIDS Terminology Guidelines available at  <https://www.unaids.org/sites/default/files/media_asset/2024-terminology-guidelines_en.pdf> and found that both “people living with HIV” and “HIV-positive” remain in use. In fact, the document explicitly lists “HIV-positive” as one of the preferred terminologies (page 25 of the guideline).  We also confirm that discouraged terms such as “HIV-infected” were not used in the manuscript. Given that both terms are acceptable, and to avoid multiple unnecessary mark-ups across the document, we have retained “HIV-positive” for consistency. A relevant excerpt from the guidelines is provided in the screenshot below for reference. Thank you.    Thank you.  We received all comments submitted by the reviewers as attachments, and each point has been carefully considered and addressed. A detailed response to every comment is provided below:  All figures meet the required technical requirements.   - As already clarified, we reviewed the 2024 UNAIDS Terminology Guidelines and found that both ‘people living with HIV’ and ‘HIV-positive’ are acceptable, with ‘HIV-positive’ listed amongst the preferred terminologies (see page 25 of the guidelines). To maintain consistency and avoid unnecessary mark-ups, we have retained ‘HIV-positive’. Thank you. - The comment appeared incomplete, making it unclear what specific improvement was requested. Nevertheless, the sentence was reviewed to ensure accuracy and that it conveys the intended meaning. - We still deleted the abbreviation here and used it elsewhere with clear explanation that it represents HIV-positive orphaned and vulnerable children aged 0–17 years. - Thank you for the comment. We have retained the acronym CLHIV but clarified in the text that it refers specifically to HIV-positive orphaned and vulnerable children aged 0–17 years (see line 30 of the track changes version). - Thank you. As advised, this has been corrected to ‘children living with HIV’ and is now applied consistently throughout the manuscript (Line 104 of the tracked changes version). - As advised, we have used the ACHIEVE acronym (see Line 126 of the track changes version). - Thank you. We have revised the sentence to indicate that “…all CLHIV were eligible to benefit, either directly or indirectly through their caregivers”, see Lines 193–194 of the track changes version. - Thank you for this observation. The referenced information has been removed from the methodology section and retained in the results section as advised. The noted slight inconsistencies arose because the figures in the methodology were reported for the overall sample (n = 26,257), whereas those in the results were presented separately for Group 1 (n = 21,448) and Group 2 (n = 4,809) (see deleted lines 201–205 in the track changes version). - Thank you for this comment. The confidence intervals were initially removed following a reviewer’s suggestion to reduce the level of detail. However, given their importance in interpreting the estimates, we have reinstated them in the manuscript (see Table 3). - Thank you for the suggestion. We have updated the wording to ‘findings suggest’ instead of ‘results indicate’ (Line 487 of the track changes version). |

END
